# Ultra-small NIR-Responsive Nanotheranostic Agent for Targeted Photothermal Ablation Induced Damage-Associated Molecular Patterns (DAMPs) from Post-PTT of Tumor Cells Activate Immunogenic Cell Death

**DOI:** 10.7150/ntno.76720

**Published:** 2023-01-01

**Authors:** Shankar Sobhana, Namratha Partha Sarathy, Laxmanan Karthikeyan, Krishnamurthy Shanthi, Raju Vivek

**Affiliations:** 1 Bio-Nano Therapeutics Research Laboratory, Cancer Research Program (CRP), Department of Zoology, Bharathiar University, Coimbatore-641 046, TN, India.; 2 Department of Biochemistry, Prof. Dhanapalan College of Science and Management, Chennai, India.

**Keywords:** Nanotheranostic, Photothermal therapy, Photoacoustic imaging, Immunogenic cell death, Cancer

## Abstract

Theranostic nanoparticles (TNPs) is an efficient avenue that culminates both diagnosis and therapy into cancer treatment. Herein, we have formulated a theranostic nanocomposite (NC) with CuS being the ultra-small core component. To ensure stability to the NC, PEI was added which is a vital anchoring group polymer, especially on sulfide surfaces, and adds quality by being a better stabilizer and reducing agent. Additionally, to add stability, specificity, and added photothermal efficiency to the fabricated NC. In addition, encapsulation of indocyanine green (ICG), an efficient NIR absorber, and Folic acid (FA) were conjugated systematically, characterized, and analyzed for photo-stability. The photothermal conversion efficiency of the novel NC (CuS-PEI-ICG-FA) was analyzed at 808 nm, where the NC efficiently converted light energy to heat energy. The NC was also tested for hemocompatibility to clarify and also determined biocompatibility. Surprisingly, damage-associated molecular patterns (DAMPs) from post-PTT of tumor cells activate immunogenic cell death (ICD) for tumor-specific immune responses. The deserving photothermal performance and photo-stability makes the NC an ideal platform for photoacoustic imaging (PAI). A superior contrast was observed for PAI in a concentration-dependent manner enhancing the level of penetration into tissues, thereby better imaging. On account of this study, the newly formulated NC could be utilized as a **“*nanotheranostic*”** designed for therapeutic and image diagnostic agent of cancer biomedical applications.

## Introduction

Photothermal treatment (PTT) is an alternative in contrast to chemotherapy, radiotherapy, and surgical procedures. With the advancement of photothermal materials, this treatment holds huge potential in clinical translation 1. PTT which utilizes photothermal agents (PTAs) to transform light to heat for the thermal ablation of cancer cells, has drawn in incredible consideration in view of specific benefits, for example, negligible intrusiveness, high explicitness, hardly any inconveniences, and low toxicity to healthy tissues. Studies have been given in the investigation of various nano-PTAs for PTT 2, 3. Therefore, cancer cells death is initiated by the heat generated in the tumor site under the exposure NIR. PTT has the potential to directly eradicate and destruct the tumor cells in the primary tumor site itself, or during local metastasis in the lymph node in order to combat the inital stages of cancer metastasis. The mechanism of PTT can also be functionalized with recent therapeutic modalities to treat the cancer cells in a highly prompt and effective manner 4. The photothermal transducer, the wavelength of the laser's light, and the method of delivering laser light all have a significant role in the photothermal absorption in tumors (either interstitial or non-invasive). PTT utilizes nanomaterials embedded in tumors as the energy absorbers in an exogenous manner which strategically convert laser light to heat source of energy 5. This is a very sophisticated method for eliminating tumors. All PTT laser light delivery techniques strive to uniformly raise tumor tissue's temperature while sparing nearby healthy tissues from harm. When the tumor temperature exceeds 41 °C, photothermal damage to the tumor cells normally starts 6. PTT frequently necessitates the tumor to achieve higher temperatures (>50 °C), and a temperature gradient will occur so that the edge of the tumor will reach therapeutic temperatures. However, efficient ablation of the tumors demands the elimination of every cancer cell 7, 8.

Specific conveyance of therapeutic agents into strong solid tumors has been viewed as one of the significant obstacles in accomplishing long haul remission and complete cure 9. Theranostic NPs (TNPs) are multifunctional nanomaterials which are planned and described for disease proficiency. They have the capacity to consolidate diagnosis and therapy in one biocompatible NPs. Somewhat recently, there has been growing interest in the improvement of different types of TNPs for cancer imaging and treatment. Viable focusing on TNPs to explicit tumor site is pivotal for both diagnosis and treatment 10-12. Theranostics helps to culminate various modalities of cancer therapy such as fluorescence, photoacoustic imaging (PAI), gene therapy, PTT and Photodynamic therapy (PDT). To modulate superior NPs with extraordinary function, the photophysical properties play a significant role. The process of understanding of various electronic transition levels in a molecule are understood in the Jablonski diagram. It helps to overcome and administer the NPs with a successful efficiency 13.

Furthermore, they should likewise have enhanced and faster clearance from the body or to be degraded to nontoxic side-effects 14. These NP highlights rely upon various properties including size and generally overall charge. Ultra-small NPs (HD size = 1-5 nm) are underneath the limit of renal disposal, while bigger NP (e.g., >20 nm) are typically cleared through the hepatobiliary framework 15. Although, numerous kinds of TNPs have been developed in the course of the most recent years, hardly any meet all the ideal standards. NPs have been made of various materials, organic nanomaterials or inorganic nanomaterials. They are carbon nanotubes, quantum dots, liposome NPs, polymer NPs and metallic NPs. Among the metal-based NPs, gold NPs (AuNP) have been the most evolved and sped up towards clinical preliminaries 8, 16.

The TNPs formulated as part of this research is a copper sulfide (CuS). It falls under the category of inorganic NPs. Enunciating the importance of why the chosen metal component Cu was utilized as the central core material is that Cu is known for its cost efficiency, easy availability, biodegradability, excellent biocompatibility, and d-d transition NIR absorption levels in peaks (600- 1100 nm) 17, 18. Accordingly, it isn't astonishing that this is additionally the compound showing the more intense LSPR band. In this regard, it merits reporting that the plasmonic pinnacle or peak of this material is naturally centered in the NIR site. This element makes Cu_2_-xS (and especially CuS) NPs amazingly vital taking into account their application for PTT and for *in vivo* applications. CuS is an excellent material that can be effectively brought to utilization in sensing, molecular imaging, PTT and drug delivery. It has emerged to be an impressive material that helps in melding both imaging and therapy 19. This greatly imposes CuS to be a multifunctional TNPs agent that has the potential to tailor depending on the demands in therapeutics.

CuS NPs have a low free carrier density when compared to other nanomaterial forms like gold-based ones, but they absorb a broad level NIR wavelength. The vital features of these CuS NPs is that they are highly stable even with a long exposure towards optical excitation, very hassle free formulation or synthesis, and display quite an analogous magnitude of heat conversion efficiency (HCE) as gold NPs. The advantages of incorporating CuS in NPs synthesis that could help to expand the biomedical outcome**.** The reason why CuS is mostly stated in comparison with gold NPs is that, previously there has been multifarious data for the photothermal capabilities of gold nanostructures. The main concern for gold NPs is that they are extensively less economical but display a broad NIR Localized Surface Plasmon Resonance (LSPR). But the major constrains are its size (above 100 nm), change in LSPR outlook on persistent optical exposure, and also alteration in HCE which is normally 80% in case of less exposure. On the contrary, CuS is quite similar and has much more efficient characteristics such as very economical, easy to synthesize, d-d transition over LSPR, free holes in valence bands which are filled by NP elements and in turn yields a high HCE and very strong LSPR absorption. These NPs can be a promising type of nanomaterial forms for various types of therapy 20. However, the use of inorganic NPs *in vivo* is restricted by toxicity. Although earlier reports demonstrated that CuS is extremely biosafe, further research is still required to determine the toxicity of CuS NPs. According to Zhou report, when PVP-coated s-Cu_2-x_S NPs were employed in a toxicity investigation and they discovered that no mice deaths occurred at injection doses of 60 mg/kg, which is 15 times higher than the effective level used in the *in vivo* PTA of 4T1 tumours. Acute blood chemistry, liver function, and renal function were not significantly affected by treatment with CuS NDs at doses up to 80 mg/kg 21. The previous studies proved that small size NPs based on CuS have no significant toxicity and it has great potential for biomedical application 22, 23.

**Scheme 1:**
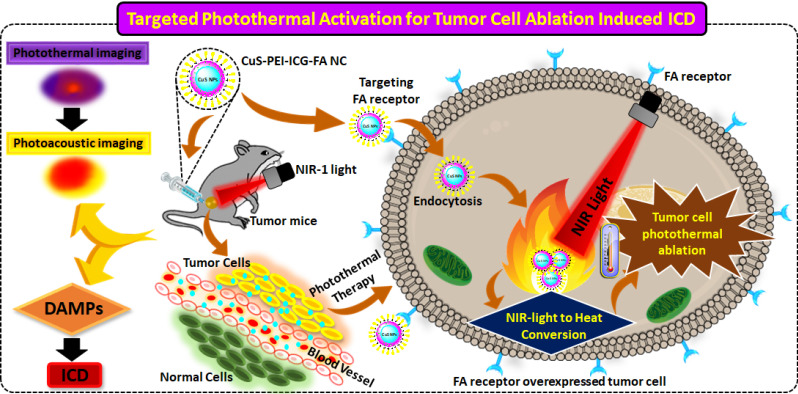
Illustration of complete conceptualized image of the complete process and working mechanism of NC induced photothermal ablation activate immunogenic cell death and imaging.

In this research study, ultra-small CuS to ensure stability of this NPs, polymers that act as stabilizing agents are employed and this can enhance the stability of the metal NPs. The polymer used is polyethyleneimine (PEI), which is an electrolyte cationic linear polymer, with a high degree of viscidity and water solubility. At varied conditions, PEI was experimentally proven to exhibit a dual property as a stabilizing agent and as a reducing agent, verifying that no aggregation takes place of the formed NPs 24. PEI stands out in various distinctive properties. It is hydrophobic, economical and is highly biocompatible. It is an efficient DNA condensing agent as it exhibits a high positive charge density which further leads to a high electrostatic interaction with the anionic DNA 25. PEI adds quality to the NPs by being a better stabilizer for two major reasons, one being a branched architecture which allows the wrapping or surrounding the core in a highly efficient way and other factor is that it contains all types of amino groups- primary, secondary, tertiary groups and thus is vital anchoring groups especially on sulfide surfaces 26. They are also known to be capping ligands for transition metals and thus form co-ordination bonds on them 27. Additionally, to add stability and specificity to the fabricated NPs, indocyanine green (ICG), an efficient NIR absorber and Folic acid (FA) were conjugated and the finally achieved a novel CuS-PEI-ICG-FA nanocomposite (NC) was designed and was progressed for further specific studies to clarify and determine the specificity of the NC. The mechanism of action of NC targeted photothermal activation induced cancer cell ablation and immunogenic cell death (ICD) activation illustrated in **Scheme 1**.

## Results

### Synthesis Process & Characterization of NC

The majority of the chemotherapeutics used today have significant drawbacks, such as the inability to deliver focused therapy and intracellular drug breakdown within the lysosomes. These explain why the average survival rate, throughout all cancer types, is still as low as 50%. This can be effectively managed under nanotherapeutic interventions. One such novel contribution to the approach is the development of the new NC (CuS-PEI-ICG-FA). The synthesis is a complete aqueous process. To obtain PEI coated CuS NPs, Copper (II) chloride solution (CuCl_2_), PEI were mixed all together in water. When CuCl_2_ is mixed with pure PEI i.e. colorless, a dark blue color change occurs due to the interaction of Cu^2+^ with the cationic polymer (PEI). The indication is a chromogenic reaction and complex formation (cuprammonium complex). This solution is allowed for magnetic stirring for 30-45 minutes at room temperature. After appropriate stirring, sodium sulfide (Na_2_S) is added to the existing stirring setup and further allowed to stir for 15 minutes. Additionally, the temperature of stirring is gradually increased upto 90 °C for 1 hour 30 minutes. Throughout the mentioned time period the brown color on the addition of Na_2_S after quite some time, on increasing the temperature changes to a dark green color. This change in color confirms the formation of PEI coated copper sulfide NPs (CuS NPs). The formed NPs are shelved in an ice cold bath at 4 °C. Once the PEI coated CuS NPs are formed, a portion of the NP solution is taken and fluorescent imaging dye component ICG is added and stirred for another 30 minutes. Later, addition of FA is done and allowed to stir for 15-20 minutes. The green coloured final solution encompassing the imaging agent and the FA have been well incorporated with the PEI coated CuS NP. Thus the final NC of CuS-PEI-ICG-FA have been successfully synthesized and was preserved in an aluminum foil wrap (due to ICG) at 4 °C. The ICG loading capacity and efficiency percentage calculated as 16.2 % and 85 %, respectively.

**Figure 1:**
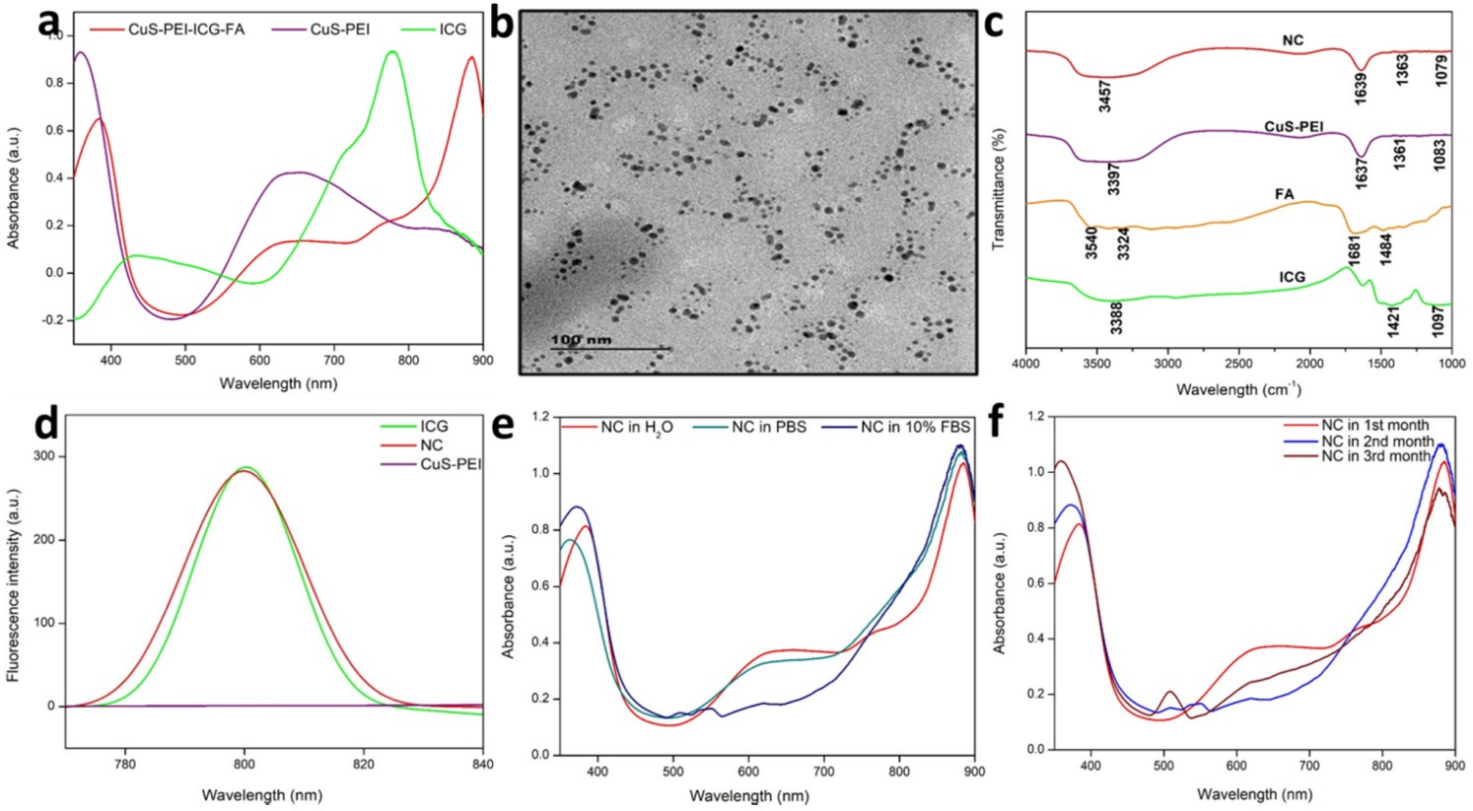
**a)** UV-vis-NIR absorption spectra of ICG, CuS-PEI and CuS-PEI-ICG-FA NPs. **b)** TEM morphological analysis for the obtained CuS. **c)** FT-IR analysis of the prepared NC, CuS-PEI, FA and ICG. **d)** Fluorescence emission spectra for ICG, CuS-PEI and final NC, with the same ICG concentration. **e)** Stability of the NC in the UV-vis-NIR absorption spectra in various biological media namely PBS, DMEM complete and incomplete and 10% FBS over an incubation period of 7 days. **f)** Stability of the NC in the UV-vis-NIR absorption spectra during the 1^st^, 2^nd^, 3^rd^ month after preparation.

A typical UV-*vis*-NIR spectra for the formulated NC of CuS-PEI-ICG-FA is shown in **(Figure 1a)**. It is an important primary confirmation for the NC and depicts an understanding towards the interfacial interaction between CuS-PEI and CuS-PEI-ICG-FA. The figure distinctively shows the absorption spectra for the NC, CuS-PEI and ICG. Since the synthesized compound is PEI coated CuS at the initial synthesis stage itself, the absorption spectra were recorded for the NC, CuS-PEI and ICG. Since PEI is a cationic polymer and has no characteristic line of absorption from 250-800 nm, to also add, it in itself acts as an excellent probe for Cu^2+^ compared to other transition metals. The recognition ability of PEI is higher and encompasses a high sensitivity especially for Cu^2+^, a simple coordinate complex formation takes place between PEI and Cu^2+^. The complex formation is a chromogenic reaction which is because of the action of Cu^2+^ on the amine group of PEI, thus forming a dark blue colored complex named “cuprammonium” complex. This complex formation in turn causes an absorption shoulder at approximately 300 nm and 630 nm. This has been evidently examined and recognized in the spectra analysis both spectrometrically and visually. The formulated NC has absorption shoulders at approximately 370 nm, 600-900 nm which are the approximate absorption regions for FA (approx. 370 nm), ICG and CuS (approx. 600-900 nm). However, the NC as such entails the NIR-I window i.e. from 700-900 nm to achieve the best of its NIR responsive therapeutic significance.

The TEM analysis is a light based imaging technique and was analyzed for the synthesized NPs to assess the grain size, morphology and dispersion. Thereby, the TEM direct observation for the NPs, enables to understand the morphological assembling of the individual constituents. The results reveal that the CuS is perfect spherical in shape and has uniform arrangement with highly dispersed in nature. The image also signifies that the CuS is very small and has an average diameter <10 nm **(Figure 1b)**. Dynamic light scattering (DLS) is utilized to figure the diameter of various sorts of particles scattered in a liquid medium. NPs in the size range 100-200 nm have been displayed to extravagate through vascular fenestrations of tumors (the EPR impact) and getaway filtration by liver and spleen. Concerning size increments past 150 nm, increasingly more NPs are ensnared inside the liver and spleen. Ultra-small-sized NPs (<10 nm) are sifted through by the kidneys. Taken together, NPs averaging ~100 nm are the most durable in the course of conveyance. The estimation of DLS investigation relies upon the size of the NPs core, the size of structural design, NP concentration, and the type of particles present in the medium. It additionally helps to understand whether the particles agglomerate over a long time by seeing whether the hydrodynamic radius of the NPs increments. On the off chance is that if particles aggregate, there will be a bigger populace of particles with a larger radius. The DLS result shows that the NC particles were well dispersed in various biological medium and the diameter in nm is around 60 nm **(Figure S1)**. This is due to the aqueous solution of CuS NC.

FTIR analysis is a versatile technique for surface characterization of NPs helps to correlate on the nature of the NP and the functional groups held in the nanosystem. It also helps to identify and assess the attachment of various compounds on the NC. It well supports the fact about the importance of optical properties of any nanosystem. It also helps to investigate if the components of a nanosystem have well adsorbed by the analysis of functional groups in final composite. In the observed FTIR spectrum, the final NC, CuS-PEI-ICG-FA, in which the core is a metal i.e. CuS that further holds PEI, ICG and FA all in a compact fabricated nanosystem. The NC has assigned vibration peaks in different wavenumbers in cm^-1^. The keenly observed vibration peaks constitute the significant peaks of all the constituents in the NP such as PEI, ICG and FA held all by the core NP. The bands attributed at 630 cm^-1^ implies to the CuS structure according to previous findings. The vibration peaks at 1363 cm^-1^ and correspond to the N-H band stretching in PEI and the strong peak at 1639 cm^-1^ aligns with the aromatic carbonyl C=O which is present in FA. The entire individual constituent's reported vibration peaks were in fair agreement with the final NC **(Figure 1c)**. Therefore, the results further lend more weight to the fact that the core has well supported the assembly of all the components to form the final innovative NC.

The fluorescence emission spectrum was also studied in order to verify the channelizing of the NC at the exact point of delivery. The emission spectra for CuS-PEI which is the primary compound, was analyzed. As already known, the primary compound is devoid of any constituent that inherits the property of fluorescence and thus shows a straight line with no emission of fluorescence. Later, after modification of the primary compound with ICG and then FA, where the fluorescence imaging dye is ICG, the final NC exhibits fluorescence. This confirms the proper anchorage of ICG with the NC as the resultant emission spectra shows a greater enhancement in the fluorescence of the NPs. Thus, fluorescence emission absorption for the PEI coated CuS, free ICG and the final NC is shown **(Figure 1d)**. The individual fluorescence spectra for ICG records a peak at approximately 800 nm and the same absorption peak is also present in the final NC. This shows an excellent binding of free ICG on the prepared NC and could definitely show good photothermal property.

Another common challenge after the synthesis of NC is to understand and analyze the stability in terms of application-associated environment. To examine this aspect of colloidal stability, the NC was tested upon commonly used biological media namely PBS, DMEM and 10% FBS media **(Figure S2)**. The results obtained were compared with NC in water, which was already obtained during the primary UV analysis. On visual inspection, no change in color was observed after an incubation period of the NC for up to 7 days **(Figure 1e)**. The reaction of PEI which acts as a colloidal stabilizer, chelating agent especially for Cu^2+^ can help in achieving better colloidal stability due to the electrostatic stabilization that takes place in the NC.

The complete process of the NC was synthesized at room temperature and they were stored at 4 °C as stated earlier. To test and analyze the reactivity and stability of the NC, periodic detection in spectrometry was carried out. The analysis was carried out during the 1^st^, 2^nd^, 3^rd^ month after the synthesis of the NC **(Figure 1f)**. The first and foremost feature in dispersion stability of the NC is that, no characteristic change in color of the prepared NC was noted at any point throughout the stage of preservation. At all months of inspection in absorption spectra, the NC showed minor changes in wavelength but still showed a persistent spectral range in the NIR-I window. This sufficiently validates the level of dispersion stability of the NC over the period of months and also substantiates that the reaction time or the duration of preservation has no significant effect on the NIR spectral range of NC.

It is also previously understood from various papers that electrostatically stabilized NPs generally may have a greater tendency to agglomerate in biological media due to neutralization of surface charges by the ionic components present in the respective media. It is also observed that the NPs adsorb the proteins in the media through simple electrostatic interaction. Therefore, this fouling away of proteins from the media by the NPs can serve to be one of the deliberate reasons for decrease in stability in terms of increased NC size and eventually precipitation of particles. This examination confirms the stability of the NC as no distinctive change in absorption band was noticed. Hence the point is that, the prepared NC seemingly has an excellent colloidal stability which maybe because of the stable and dense hydrophilic PEI coating over the CuS NP. This study was done in order to add value to biomedical applications of the prepared NC. The NC exhibited a fair level colloidal stability in terms of color and also no particle precipitation was further noted during the course of preservation for 1 week. The Energy dispersion X-ray (EDX) analysis for the NC in **Figure S3** indicates that the atomic ratio of Cu to S was close to the stoichiometry of CuS. Their element mappings illustrated the homogeneous distribution of Cu and S elements in the CuS frame work **(Figure S4)**.

The zeta potential, which relies upon the surface charge, is significant for the steadiness of NPs in suspension and is additionally the major point in the underlying adsorption of NPs onto the cell membrane. After adsorption, the endocytic uptake rate depends upon the molecule size. Charge adjustment of nano-frameworks; offers an opportunity for prolonging the blood flow time, upgrading the chance of its interaction with target cells of interest, and changing the pharmaceutical properties and effect of survival of nanosystems. The NC developed has an outer layer of FA which comprises a negative charge (-6.41 mV) **(Figure S5)** has been reflected onto the zeta analysis shown. Thus the NC formation has been well assembled among the individual components of the nanosystem.

### Photothermal Conversion and Photo-stability of NC

**Figure 2:**
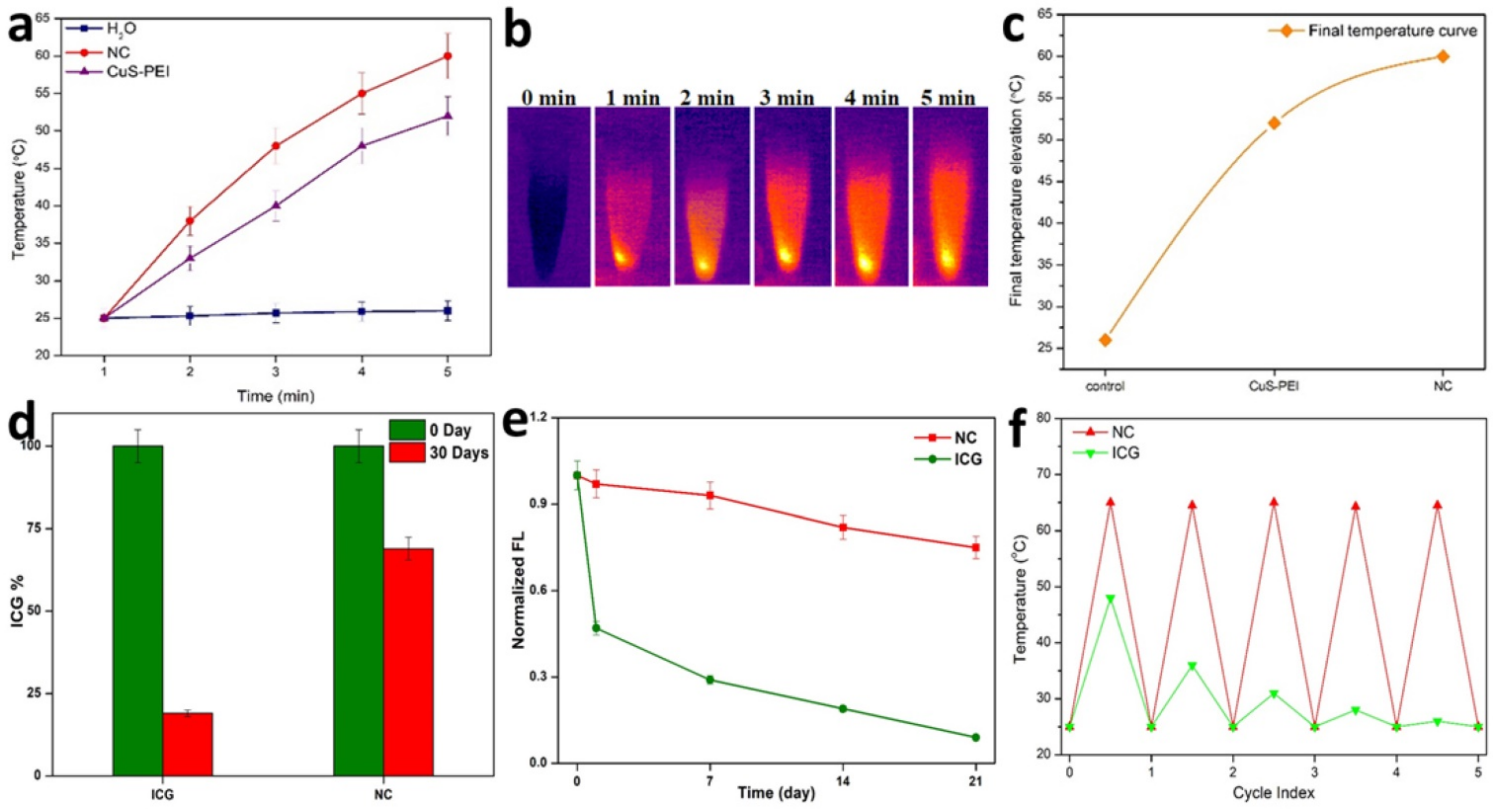
**a)** Photothermal performance of the NC, CuS-PEI, and water for temperature change profiles of different time periods from 1 minute to 5 minutes. **b)** Photothermal images of NC after NIR light irradiation by 808 nm laser for 5 minutes. **c)** Graph depicting the photothermal performance of the NC of the final temperature curve of control, Cu-PEI, and NC in 5 minutes. d) The storage stabilities (%) of free ICG and NC incubated at 4°C in the dark condition over periods of 30 days. **e)** The fluorescence intensity changes of ICG and NC in an aqueous solution for 21 days. **f)** Temperature change for free ICG and NC over 5 cycles of repeated laser irradiation.

A pivotal component in the investigation of PTT is checking the photothermal performance of the prepared NC. The temperature changes of the NC in aqueous solution and in water were noted. The observation was done at 808 nm using a thermal imager. The results showed that there was no significant temperature increase in the NC in pure water. Later, the NC was tested for photothermal performance under the wavelength exposure of 808 nm **(Figure 2a)**. There was a significant increase in temperature. Highest concentration i.e. 100 µg/mL was taken and persistent NIR exposure was given for different time period from 1, 2, 3, 4 and 5 minutes. It was noted that the temperature gradually increased as the time of exposure with NIR increased with a power density of 1 W/cm^2^. A highest temperature of 57 °C was attained at a time period of 5 minutes**.** Therefore, from the obtained confirmations, the photothermal conversion efficiency (PCE=32%) performance of the NC was well understood and previous illustrations already state that temperatures nearing 50 °C or above successfully encourage coagulative cancer cell necrosis and irreversible cell death. Previously our NC has also exhibited good stability in various biological media and thus has shown to be better photothermal agent for PTT. A significant elevation in the highest temperatures in a time period of 5 minutes is observed. It was clearly affirmed that the NC with incorporated ICG has sufficiently increased the photothermal performance of the NC, these observation was done at 808 nm using a thermal imager **(Figure 2b)**. The final temperature elevation of control, CuS-PEI, and NC were represented. The illustration through a graph shows the photothermal performance of CuS-PEI and CuS-PEI-ICG-FA NC in comparison with the control (water). The graph shows that PEI coated CuS showing an increase in temperature with 52 °C at a concentration 100 µg/mL. The graph shows higher photothermal performance. However, the NC since incorporation with ICG, which in itself has photothermal properties, shows a rapid increase in temperature with above 60 °C at a concentration 100 µg/mL **(Figure 2c)**.

Furthermore, the storage stability of ICG could also be increased by encapsulating it within NC. The storage stabilities of ICG alone and ICG loaded NC were studied after 30 days of storage at 4 °C in the dark. Fascinatingly, we found that NC showed less degradation after 30 days of storage at 4 °C in the dark condition and ICG alone showed the highest degradation rate in the same conditions **(Figure 2d)**. Thus, these results suggest that the long-term stability of NC is an advantage for its use in biomedical research applications. The fluorescence stability of ICG alone and ICG loaded NC. After 21 days of storage, the fluorescence intensity of ICG alone decreased from its original fluorescence intensity rate, whereas the NC maintained its high value. It is obvious that the fluorescence stability of ICG was improved after being loaded with NC **(Figure 2e)**. This was further confirmed when ICG lone and ICG loaded NC were subjected to NIR laser irradiation for 5 cycles, the ICG alone gradually diminished over the period of 5 cycles, but the NC exhibited unchanged photothermal stability. Therefore, the NC bearing ICG was highly stable and the conjugation of free ICG with the NC has effectively increased the photothermal stability of ICG, thus enabling better photostability to the NC **(Figure 2f)**. Therefore, the prepared NC shows a rapid increase in temperature and confirms to be a good photothermic component.

### *In vitro* Biocompatibility and Targeted Photothermal Effect of NC

**Figure 3:**
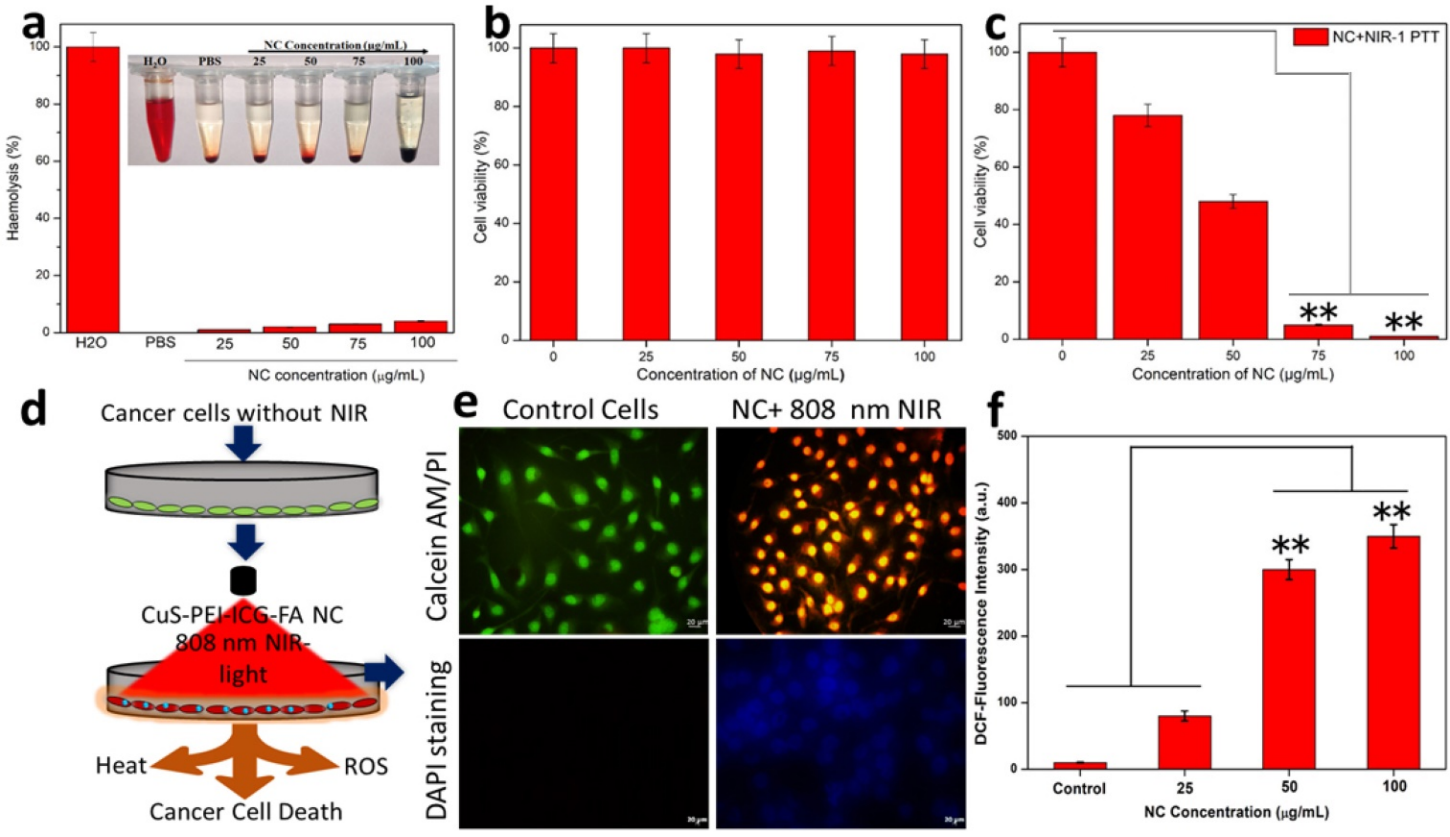
** a)** Haemolysis percentage of RBCs incubated with NC at various concentrations 25 µg/mL, 50 µg/mL, 75 µg/mL and 100 µg/mL for 24 h, using deionized water and PBS as the positive and negative control, respectively. **b)** MTT assay for biocompatibility test of the prepared NC without NIR. **c)** MTT assay for the photothermal destruction of HeLa cells with NC of different concentrations under 808 nm NIR exposure. **d)** Schematic image of *in vitro* HeLa cancer cell ablation with the NC after NIR illumination under 808 nm. **e)** Calcein-AM and PI, the cell nuclei were stained with DAPI. **f)** DCF-staining methods of quantitative results of ROS detection in HeLa cells after treatment.

According to the fundamental concept, all the materials enter the blood stream and come in contact with the red blood cells (RBCs). The hemolytic activity of most of the NPs is concentration, size, shape and structure dependent. The effort of analyzing the hemolytic effect of the engineered NPs is an essential part of preliminary assessments to evaluate the interaction of the NP with the components of blood. To evaluate the impact of the developed NPs, hemolysis test was carried out by a spectrophotometric measurement of hemoglobin release after the exposure of a series of various concentrations of NC. At the process of hemolysis, the performance of the assay was tested with the help of PBS, which was used as the negative control and water being the positive control. The NC showed a steady potential during the concentration dependent analysis. It was noted that at all concentrations namely 25, 50, 75 and 100 µg/ml the percentage of hemolysis was merely less than 5% only **(Figure 3a)**. This caters a suitable hemocompatibility with the RBCs and shows good blood compatibility at various concentrations of NC **(*inset* Figure 3a)**.

The effect of the prepared NC in the selected cell line for this study (HeLa cells) was carried out. This vitality is of essential importance to understand the potential of the NC especially in the biomedical applications. To ensure the biocompatibility and photothermal effect of the NC, it is primarily important to showcase its biocompatibility with the cells, thus endorsing the non-toxicity and high biocompatibility with the selected cell line. Firstly, the NPs were tested for biocompatibility and henceforth the viability of cells was assessed. In terms of biocompatibility, the NC without the exposure of NIR showed almost an extraordinary rate of viable cell percentage in a concentration-dependent manner (0, 25, 50, 75 and 100 µg/mL) **(Figure 3b)**. This biocompatibility of NC is the one of the major concerns before the exposure of NIR in order to trigger the photothermal effect.

As part of this study, the NC under the exposure of 808 nm NIR was carried out in a concentration-dependent manner for 5 min. The assessed concentrations were 0, 25, 50, 75 and 100 µg/mL. On significant exposure to NIR the NC exhibited characteristic changes in a concentration-dependent manner. The NC concentrations 25 and 50 µg/mL showed a significant level of decrease in cell viability percentage approximately 75% and 45%, respectively. Whereas, the NC concentrations of 75 and 100 µg/mL under NIR-light on HeLa cells showed approximately 5% and almost complete decrease in the percentage of viable cells and are noted to be the highly significant levels of selective PTT effect **(Figure 3c)**. This shows effective photothermal ablation of the NC in the displayed cells under the exposure of NIR. Furthermore, to determine the power density of the NIR light onto the HeLa cells treated NC so as to achieve efficient cancer cell ablation was also carried out. For this study, various power densities of NIR were taken (0. 25, 0.50, 0.75 and 1 W cm^-2^) and the cells were checked accordingly **(Figure S6)**. The power densities 0.25 and 0.50 W cm^-2^ showed a considerable decrease in cell viability (%) and were observed as significant levels of photothermal effect on the cell. However, the power densities 0.75 and 1.00 W cm^-2^ showed a drastic decrease in cell viability (%) featuring almost complete power density-dependent cell ablation. Thus the ideally 0.75 and 1 W cm^-2^ is determined to be the highly significant levels of photothermal ablation of the cells. Furthermore, to obtain an effective cellular uptake intuitive evidence, tumor cells were further incubated with NC, then intracellular Cu content was detected by the inductively coupled plasma mass spectrometry (ICP-MS) to evaluate the targeted endocytosis was obtained. The NC cell internalization Cu content was studied after NC treated cells **Figure S7**, shown that expected NC uptake might be attributed by FR-positive cancerous tumor cells increased remarkably stronger intracellular uptake of NC by receptor mediated endocytic pathway.

Further, the HeLa cells were stained in order to view the effect of the NPs on treated cells. It is already known that FA has a strong affinity for the FR in the cell membrane. It is mainly for this reason that various nanocarriers are subsequently modified to improve the drug delivering capacity to cancer cells that possess the presence of overexpressed FR. The NC surface with FA must definitely help with the mechanism of total accumulation of the NC onto the target cancer cells. Then, cells were effectively double stained with Calcein-AM and ropidium Iodide (PI) staining method. In this type of staining, it is stated that calcein-AM stains only viable cells. On the contrary, the PI which is generally a nuclei staining dye cannot intrude through the viable cell membrane. The control cells remained undisturbed and cells were effectively stained green since they are viable. The cells with the NC on the other hand under the exposure of NIR at a power density 1 W cm^-2^ for a time period of 5 minutes, showed that the cells effectively absorbed propidium iodide (PI) (only ablated cells can uptake PI) and exhibited red fluorescence and almost ablated all the cells **(Figure 3e)**. It was expected that once the NC entered the cancer cells, the temperature of the cancer cells may be dramatically raised following the NIR laser's exposure, leading to causing injury to the DNA, proteins, and other substances. To assess DNA damage, fluorescent labelling with DAPI was used to analyse the sample. The cancer cells treated with NC revealed an elevated blue fluorescence, indicating DNA damage, double-strand breaks, which confirmed the critical damage to the cancer cells nuclear DNA **(Figure 3e)**. Fluorescence intensity after NIR exposure is increased also at the same concluded that NC into the cancer cells produced more nuclear damage under laser irradiation. The above results suggested that CuS-PEI loaded ICG NC induced significant photothermal heat to activate irreversible damage to tumor cells. This process for PTT response of NC was accompanied by generation of ROS simultaneously. As shown in **Figure 3f**, the fluorescence intensity of ROS generation from NC increased with post-treatment of the laser irradiation, demonstrating continuous generation of singlet oxygen by NC. Therefore, these results confirmed that ICG incorporated CuS could be used as a phototherapeutic agent for simultaneous PTT/PDT therapy activated by NIR laser. Therefore, the synergistic PTT/PDT effects induced by NC under NIR laser irradiation could efficiently convert the NIR to heat and simultaneously ROS for enhanced phototherapy.

### FACS Analysis and Photoacoustic Imaging (PAI) of NC

**Figure 4:**
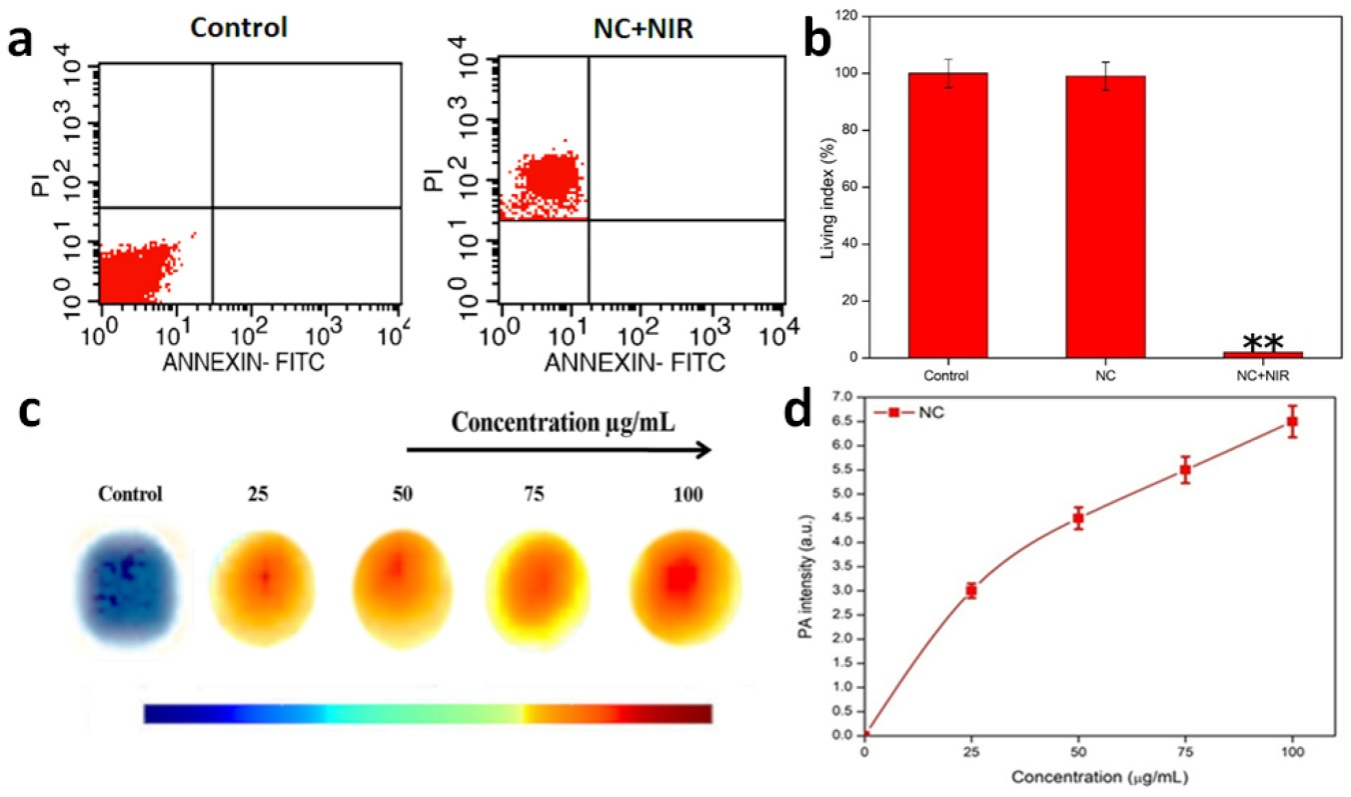
** a)** FACS- Assays of NC with 808 nm NIR light induced necrotic cancer cell ablation. **b)** Concise representation of the living index of the cancer cells in control, NC and NC under NIR exposure. **c)** PA intensity of NC and **d)** Graph representing PA intensity measurement of NC at various concentrations.

An apoptotic cell shows the large number of morphological and biochemical highlights, which fluctuate contingent upon the stimuli and cell type. The gross larger part of traditional apoptotic trademarks can be quickly inspected by flow and imaging cytometry. Necrosis and apoptosis are two significant, morphologically and mechanistically distinct modalities of cellular ablation portrayed by cell membrane obstruction and DNA fragmentation/film phosphatidylserine exposure without cell membrane destruction, respectively. Cell ablation with NC was effectively determined by measuring the cellular uptake of PI (Sigma-Aldrich) after treatment with NC for 24 hours in 100 µg/mL concentration. In general, live cells are impermeable to PI or PI cannot intrude into the viable cell membrane, and PI uptake was used to quantify the population of cells in which membrane integrity was lost. To assess on the quantification of cell death, FACS analysis was performed **(Figure 4a)**. It was interesting enough to note that, the NC treated HeLa cells took up PI in a concentration-dependent manner. There was a gradual increase in the number of cells taking up PI with respect to 100 µg/mL concentration of NC. There is a qualitative and relative change in the number of dead cells after the treatment with NC at the expense of NIR. To concisely understand, the cell viability is maintained in the case of control cells, when NC was gradually introduced in the cells, there was a fair maintenance in the living cell index, but when the cells under the exposure of NIR with the NC, there was merely few cells maintained and showed most of the cells were ablated. This is precisely depicted in the graph shown below in terms of living index percentage **(Figure 4b)**.

PAI is based on the concept of measurement of electromagnetic energy into acoustic pressure waves. During this process of PAI, the tissue is successfully irradiated with a nanosecond pulsed laser, which helps in ultrasound wave generation when optical absorption or thermal expansion of the respective tissue takes place. This process helps in deep penetration into the tissues and enhances the imaging property of NPs for cancer therapy. The photothermal performance and excellent photostability of the NC make it ideal platform for PAI. Thus, the sample concentrations of 0, 25, 50, 75, and 100 µg/mL were taken for the PAI observations. It was noted that the acoustic intensities ranged with increasing concentration. Pure water was used as a control. Gradually development of acoustic intensities was noted from 0, 25, 50, 75 and 100 µg/mL in comparison with control i.e. pure water. Considering concentration to be a determining factor of PAI, it was noted that a huge shift in intensity occurred at 100 µg/mL, offering a superior acoustic contrast in comparison with control **(Figure 4c & d)**.

### NIR-PTT-Induced Tumor Inhibition and Release of DAMPs

**Figure 5:**
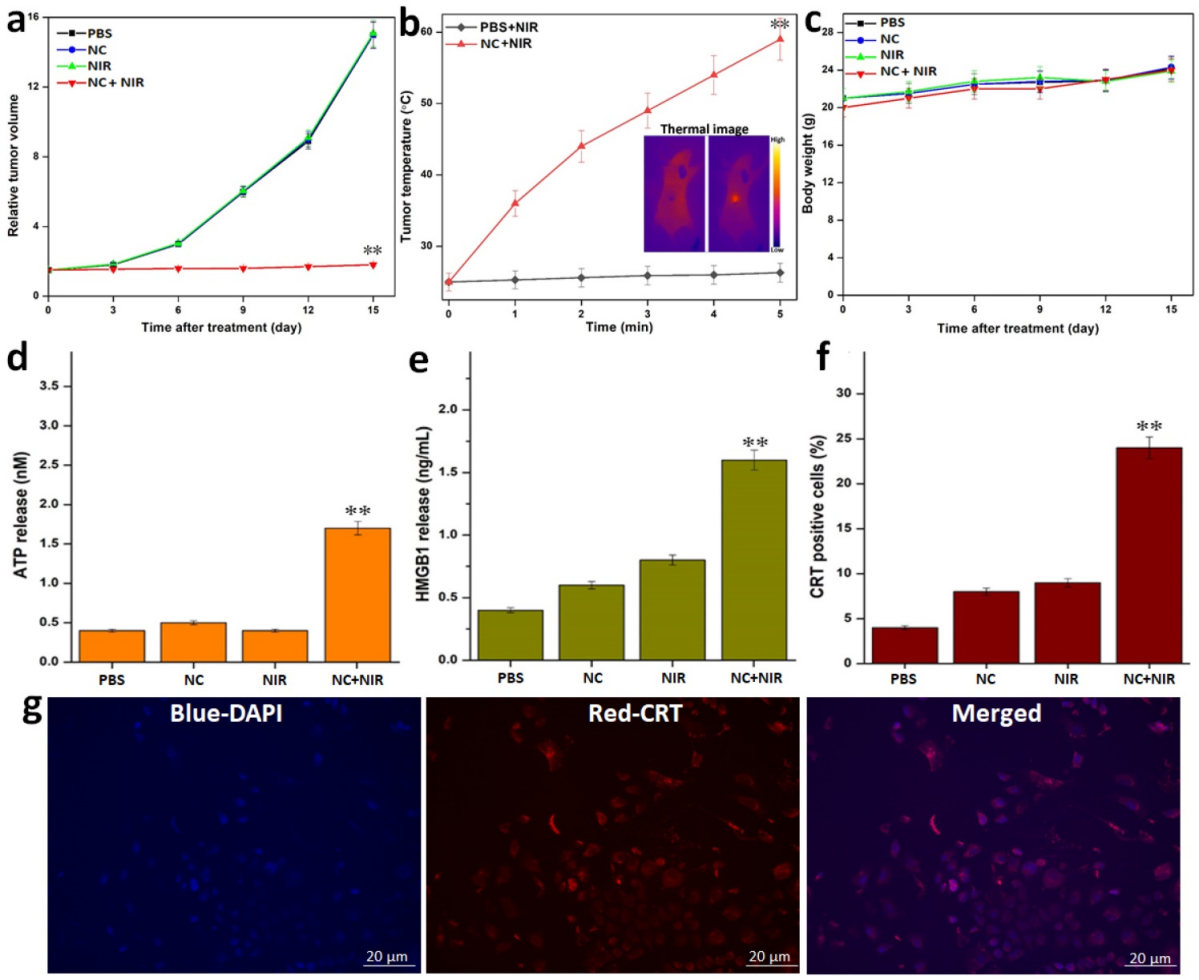
*In vivo* tumor mice with various treatments:** a)** Tumor volume growth curves for the various treatments. **b)** Temperature rise profiles of tumors and representative IR thermal images (inset figure) of tumor-bearing mice with PBS and with NC injection under NIR irradiation. **c)** Change in the body weight of tumor-bearing mice for the various treatments. NIR-I PTT induced release of ICD biomarkers of DAMPs from the tumor cells *in vitro*. **d)** ATP release by the tumor cells was detected 24 h post-PTT at different treatments. **e)** HMGB1 release from cell the nucleus to cytoplasm of tumor cells measured and quantified by ImageJ treated with PTT. **f-g)** *In vitro* CRT exposure by tumor cells in response to PTT treatment as determined, detected after post-PTT. Data are shown as mean ± SD (n=3) *p < 0.05, **p < 0.01.

Additionally, the tumor volume and tumor temperature profile with and without injection of NCs was monitored in real-time using an IR thermal camera to show the impact of NC on tumor PTT *in vivo* **(Figure 5a, b)**. The tumor development volume curves for the 4 various groups of treatment regimens over a 15-day post-treatment period are presented in **Figure 5a**. When NPs were injected, NIR imaging of the tumor volume revealed no appreciable differences in tumor growth from the untreated PBS control. However, at the same dose, NIR-treated NC tumor mice showed a highly substantial tumor growth inhibition rate that was comparable to the other groups receiving control radiation. NIR radiation was applied to tumor-bearing animals to synergistically inhibit tumor development and tumor ablation caused by PTT-induced heat production from NCs. By the time of the follow-up period, the tumors in this PTT group had been entirely eliminated with no signs of regrowth. As a result, after 5 minutes of 808 nm NIR irradiation, the temperature of the post-treated tumors with NCs increases fast. The tumor, however, only reached 26 °C when PBS alone was injected under the identical laser exposure circumstances **(Figure 5b)**. Additionally, the animal body weight of each treatment group was noted as a measure for determining systemic toxicity **(Figure 5c)**. However, no such significant reduction in body weight loss was seen across all treatment groups, showing that all treatment group animals tolerated the injection of NCs along with 808 nm NIR irradiation without any harm.

Activation of Immunogenic Cell Death (ICD) was seen following PTT post-treatment. As a result, we next looked into whether or whether post-PTT in various treatments could cause the release of DAMPs from dying tumor cells, which is a crucial ICD property. After being pre-incubated with NC, the tumor cells were exposed to 808 nm laser radiation (1 W cm^-2^). First of all, we discovered that adenosine-5′-triphosphate (ATP) molecules generated from dying tumor cells can function as a “find-me” signal and result in DCs producing cytokines. After the post-PTT, we saw an increase in extracellular ATP, which was followed by a progressive rise over the following 24 hours **(Figure 5d)**. After that, we looked at whether the high mobility group box 1 protein (HMGB1) secreted by tumor cells that were dying after PTT could act as a catalyst for inflammation, draw in different immune cells, and induce DC maturation. We discovered that, following laser irradiation, the post-PTT therapy also dramatically accelerated the translocation of HMGB1 from nuclei to extracellular space **(Figure 5e)**. A robust “eat-me” signal and mediator of tumor immunogenicity, calreticulin (CRT) exposure is the next ICD biomarker. A post-PTT dependent exposure of CRT that was strongly manifested following the post-PTT therapy was discovered by CRT measurement. The PTT caused by the 808 nm laser showed the greatest exposure of CRT caused by post-treatment, which was further supported by observation **(Figure 5f, g)**. In addition, the development of the ICD biomarkers for the aforementioned DAMPs in the dying tumor cells suggested that post-PTT treatment would be able to trigger temperature-dependent ICD. Since the NC's core CuS size is significantly smaller than the renal clearance threshold (~6 nm), one can anticipate that the particles are primarily excreted.

Additionally, we examined the blood retention profiles following the post-injection and reported that our compound has the ability to prolong NC's blood circulation. **Figure S8** shows the half-life of NC in blood at different time intervals, which demonstrates the prolonged circulation period. As a result, we hypothesized that removing PEI from CuS at the tumor location would reduce CuS blood flow and further cause the aggregation of CuS surrounding the tumor, which would result in the enrichment of CuS in the tumor. The biodistribution of NC was observed on tumor-bearing animals to support this theory. The average fluorescence of the tissue mass was used to express the NC biodistribution, which was found in several tissue samples (a.u). According to **Figure S9**, the ICG content in the tumor reaches its peak at the tissue following post-injection, indicating a significant tumor accumulation ratio. Upon post-injection, NC actively accumulated into tumor tissues as a result of the normal EPR effect **(Figure S10)**.

### Long-Term Toxicity Analysis of the NC

**Figure 6:**
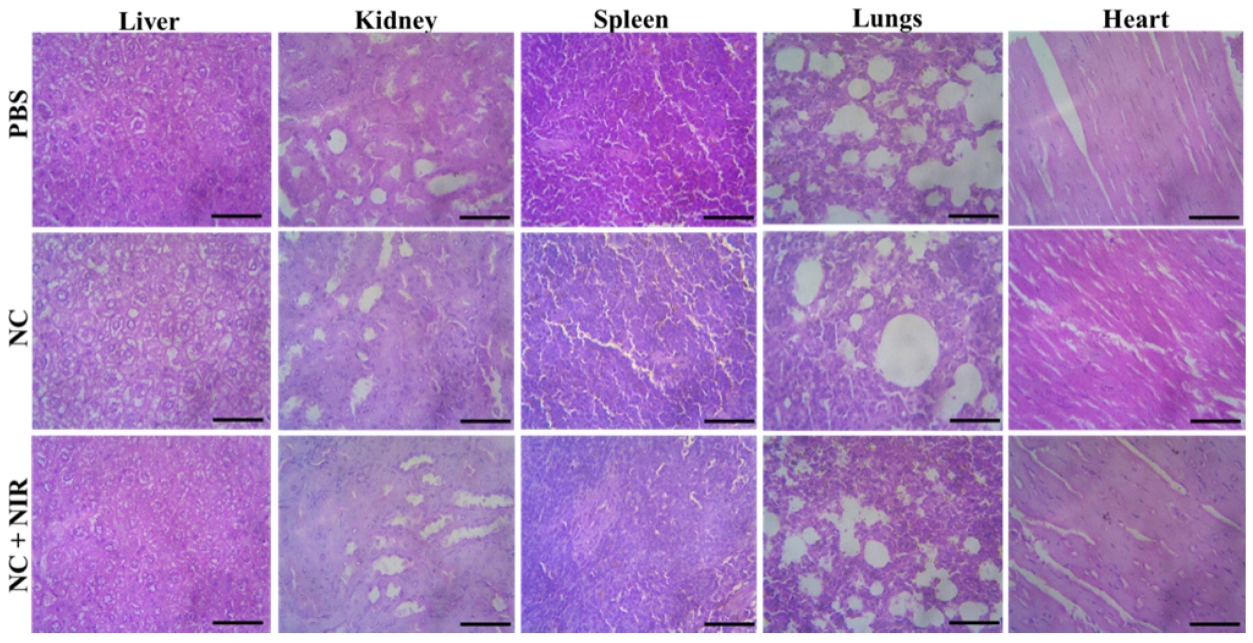
H&E stained tissues of liver, kidney, spleen, lung and heart of mice from the PBS, NC and NC + NIR groups. Scale bar 100 µm.

Then, we conducted a systematic examination on the NC *in vivo* toxicity analysis in order to assess its biosafety. At 15 days after injection, the mice's major organs including the liver, kidney, spleen, lung, and heart were removed from each group, and each organ was then histologically examined. According to H&E staining, none of the treatment groups with or without NIR irradiation showed any discernible organ damage or evident inflammation or necrosis, as shown in **Figure 6**. As a result, these histology findings from numerous organs showed that the NC had no negative effects on the major organs and that they were extremely biosafe.

**Figure 7:**
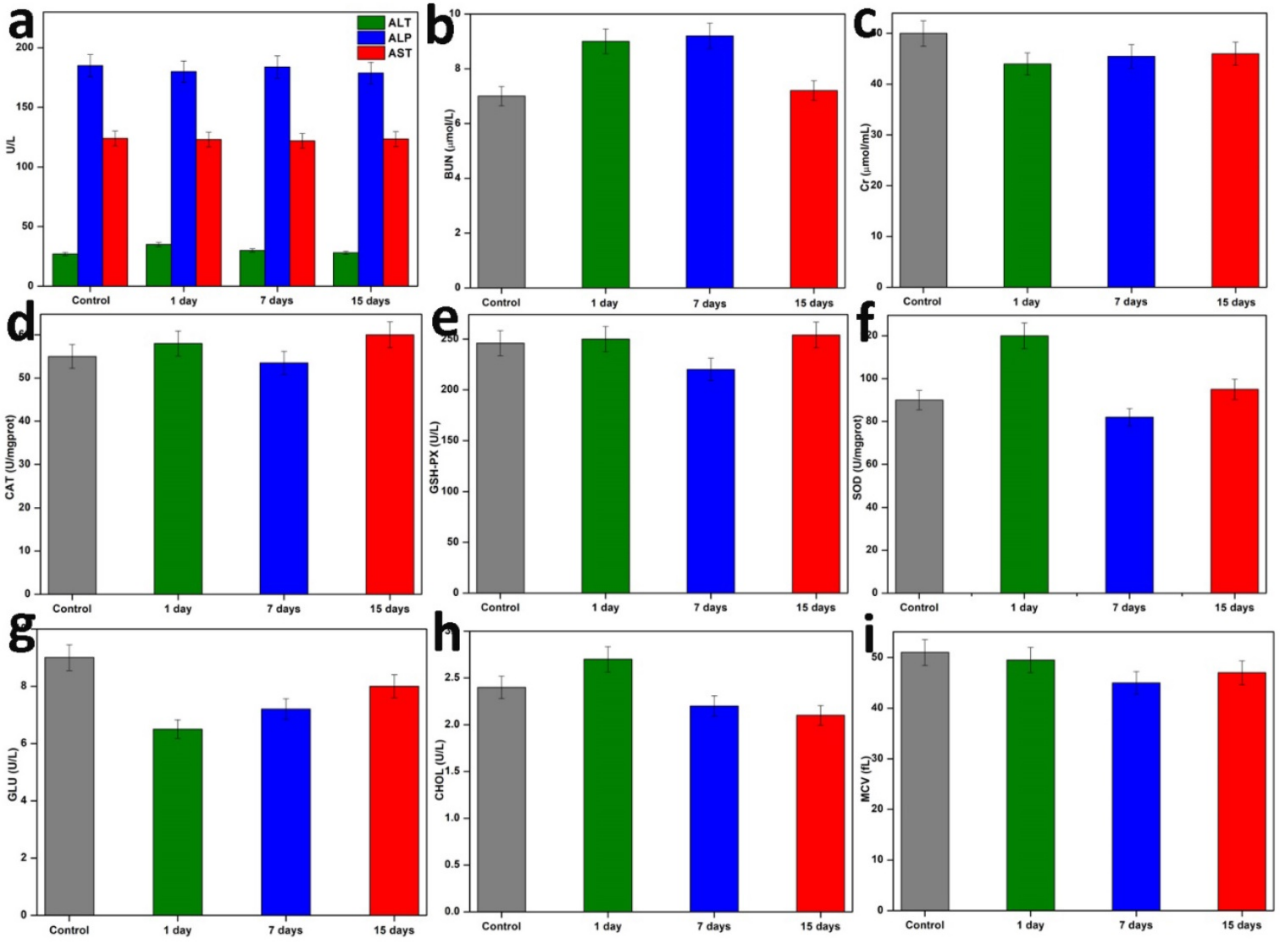
Hematology and blood biochemical assay *in vivo*. Blood indexes of ALP, ALT, AST, BUN, Cr, CAT, GSH-PX, SOD, GLU, CHOL, and MCV from the mice treated with NC and PBS at various time points (1^st^day, 7^th^ days, and 15^th^ days). Data are presented as mean ± SD.

Additionally, the blood biochemical analysis (n=3) was performed on the first, seventh, and fifteenth days following therapy. The outcomes are shown in **Figure 7**. Following the injection of NC, these parameters' levels initially changed very little, but they quickly stabilised over time. Then, it was discovered that the haematology parameters (blood glucose (GLU), blood fat (CHOL), and mean corpuscular volume (MCV) were not significantly different within normal reference ranges. The liver function markers (alanine aminotransferase (ALT), alkaline phosphatase (ALP), and aspartate aminotransferase (AST) and kidney function markers (serum urea nitrogen (BUN), creatinine (Cr), catalase (Cat), these findings revealed that NC did not cause any blatant toxicity to the liver or kidneys throughout treatment, or any other negative side effects. Based on the aforementioned parameters, we were able to demonstrate that the targeted PTT nanoplatform developed in this study had good biocompatibility and exciting potential for *in vivo* applications.

Thus, a thorough description of the entire process of NC creation and its mechanistic impact on research at the *in vitro* and *in vivo* levels is provided. Since the outer surface of the NC is made up of FA, it is easily linked to the surface FR following production. The bulk of cancer tissues have excessive levels of the FR, a glycosylphosphatidylinositol-anchored cell surface receptor, whereas healthy tissues and organs only have minimal levels of the receptor. Epithelial, ovarian, cervical, breast, lung, kidney, colorectal, and brain cancers have significantly overexpressed FR. FA is non-immunogenicity and capacity to continue binding to the FR even after subsequent conjugation or integration with medications or other targeted moieties make it an intriguing substance. The endocytotic pathway is used to absorb folate after it binds to the caveolae's receptors. Finally, the NC is released as the folate separates from the receptor. Later, the NC is activated at the expense of NIR light, allowing for the efficient ablation of cancer cells and ICD in the cancer location. The prevention, detection, and treatment of cancer are all being investigated using nanotechnology. Methods that could make cancer detection and therapy non-invasive and focused on tumors are being considered and tested. The NC ability to target only tumor cells by PTT and ICD may prove to address and overcome the adverse side effects of conventional cancer treatment.

## Discussion and Conclusion

CuS TNPs semi-conductors have been broadly investigated for their multifunctional properties 28. They have additionally arisen as promising and versatile specialists for cancer theranostics as a result of their diverse diagnostic and therapeutic potential 29-31. Among different inorganic materials, this NP is drawing in most extreme consideration due to biocompatibility, low toxicity, and cost effectiveness 29, 32. Unlike gold nanostructures and carbon nanotubes, an exceptional feature of CuS NPs is that their NIR absorption is derived from its characteristic d-d transition of Cu^2+^ ions. The primary factor of why this is to be keenly observed is that, the NIR absorption is not hindered by any solvent interaction or the influence of the surrounding environment at the advent of formulation or administering it *in vivo* 33, 34. CuS is one of the most studied transition metal sulfides for interesting properties and features. CuS has excellent PTCE when in aqueous dispersion under laser irradiation at 808nm. Therefore, it proves the importance and significance of employing these NPs as effective PTAs for ablation of tumors 35-37. In the initial preparatory state, the donor for copper chloride (CuCl_2_) used is sodium sulfide (Na_2_S) which is one of the frequently used donors to obtain CuS NPs. CuS when formulated as the core has extraordinary aspects related to drug delivery and nanotherapeutic efficacy. It is characterized by high molar extinction coefficient, considerable PTCE, remarkable NIR optical absorption and high metabolizing ability in humans 28, 38. The irradiation of CuS NPs is that the process of irradiation allows the gating of local heating mechanism and efficiently produces a strong photo acoustic signal. This is another milestone with respect to CuS NPs which are proving them to be diverse nanomaterials in cancer therapy 39.

To substantially increase the efficacy and stability of the NP, other constituents were periodically added along the process. As part of the designed work, the NC synthesized was CuS-PEI-ICG-FA. The UV optical absorption spectra showed the absorption bands of the NC. The peaks shouldering at around 300 nm and 630 nm are the bands due to the formation of cuprammonium complex. This is a bright blue colored complex developed due to the interaction of Cu^2+^ with PEI; the blue color can vary with intensity with respect to the concentration of Cu^2+^ ions. This is one of the preliminary confirmations for the mechanistic reaction between Cu^2+^ and PEI which is a typical chromogenic confirmation 40. The NC showed excellent absorption peak shouldering between 600-900 nm approximately and shows pronounced photothermal potential in the NIR-I window (700-900 nm) **(Figure 1)**. A similar result in the optical studies (UV-*vis* NIR absorption spectrum) for the complex Cu^2+^-PEI has been reported by Wen *et al*., 2014. It is also noted that the broad band of UV attained in our study from 600 to 900 may be due to the abundant amine groups present in PEI which on interaction with Cu^2+^ i.e. the cupric amine complex 41. In another study by Bharathiraja *et al.,* 2017, CuS NPs were incorporated with chlorine e6 (Ce6) for the targeted ablation of cancer cells. Herein, PEI was used as high density amine group polymer, to film on the CuS NP after which the NP was incorporated with Ce6. According to the study, the fictionalization with PEI greatly enhanced the absorption of CuS up to 830 nm, since colorless PEI alone has no characteristic absorption bands. The cellular internalization of the NP was understood and was reported that 200 µg/ml NP showed a cell viability of 92.5% and 60% cell death was achieved with the same concentration of NPs 42.

The NC developed by us was tested for stability under various conditions, one of which was analyzing the absorption spectra at various physiological media. It was noted that the samples did not show any distinctive change in the visual analysis i.e. any aggregation or precipitation was not seen in the samples. Then the samples were subjected to UV analysis, where there was no wide alteration in the absorption spectra of the NC and the NIR range window I remained as such, which therefore shows the foundational stability in the NP **(Figure 1)**. Dos Santos *et al.,* studied thioglygolic acid coated (TGA) CuS nanofluid (NF), the whole process was a one pot synthesis that brought about a stable NP formulation that was stable in aqueous media and had high colloidal stability and dispersive capability. Thus the NF showed an excellent heat exchanging properties and a thermal conductivity of 38% 43. Another surface-ligand approach by Ding at al., with PEG coated CuS NPs were formulated. These NPs were designed due to the reason that synthesizing CuS NPs with enhanced colloidal stability and highly tunable size is a challenging one. It was positively observed that the NPs showed less size variations and agglomeration of the samples was also not detected 44.

The approach of multifunctional TNPs serving both diagnostic and therapeutic abilities is given special attention in order to improve the therapeutic outcome of any NC. Developing NPs with certain well performing contrasting agents to identify and understand the lymphatic drainage of the tumors. Therefore, an exogenous agent like ICG, a fluorescent dye is an innovative imaging modality when combined with NPs. The fluorescence studies reveal that the intensity was greatly upgraded after the uptake of ICG and thus the fluorescence capacity of the NC could enable to hold value for the image guided therapy and the visualizing of tumors in an efficacious way. Li *et al.,* developed a strategic design of a nuclear targeting CuS based NP which was encapsulated by oporous silica and then modified by TAT peptide, for the efficient targeting of the nuclear pore complex. The NPs CuS@MSN-TAT was further modified by Rhodamine (RHB). The fluorescence spectra after the attachment of RHB significantly increased stating the proper anchorage of the component on the core NP 45. Another study by Li *et al.,* was developed to carry two antitumor drugs namely, Doxorubicin (DOX) and chlorin e6 (Ce6) where Ce6 is a fluorescent marker used in many cancer studies. It also enables the formation of reactive oxidation species (ROS) and thus serves to be an excellent component of photodynamic therapy (PDT). Here, hollow CuS (H-CuS NPs) which were combined with a phase change material (PCM, 1-tetradecanol) which was already loaded by drugs. The NC (HPDC NPs) was a combination of all these essential modalities that turn out to be multifunctional. Thus the NC fulfilled biosafety with a PTCE of 44.13% which was considerably good for successful ablation of targeted tumors in the target site 46. This highlights on the image guided approach that can be modulated with the help of CuS NPs. There are a number of advantages and myriad research studies ongoing to investigate and modify CuS NPs for cancer therapy apart from the widely reported Au nanostructures 28.

Liu *et al.,* devised a novel attempt by developing cystein-coated CuS with a highly proficient PTCE of 38%. It was also noted that the biocompatible nature of CuS was especially due to the cystein coating and could withstand on stability on its composition by 1 week. In order to observe the PTT and the feasibility of the NP, the NPs were tested upon HeLa cells. It was understood after the analysis that the Cys-CuS NPs showed exceptional viability over the HeLa cells even at the maximum concentration of 75 ppm 47. Therefore, the developed novel NC also encloses excellent biocompatibility, better PTCE, excellent hemocompatibility **(Figure 3a)** and targeted photothermal efficiency under 808 nm NIR light exposure **(Figure 3c and e)**. Another interesting conjugation of CuS was done with fucoidan by Jang *et al.*, to effectively work three cell lines HeLa, K562, A549. Fucoidan is believed to have interesting features when it comes to cancer therapy. It was shown under speculation that F-CuS NPs at 50 µg/mL and 100 µg/mL showed effective and complete cell ablation of HeLa cell lines at 808 nm laser for a time period of 5 minutes 48. Various capping agents add more biomedical value to the formed NP. They act as protective shields in conferring the core with longer circulation time and effective targeted delivery. In another study by Mofokeng *et al.,* alanine was used as a major capping agent to CuS NP and they were tested upon HeLa cells. It was noted that the NPs exhibited decreased cell viability in a concentration dependent manner. The NPs were not toxic in low concentration levels 49. Hence, the present research study reported that CuS not only attributed to be a photothermal agent but also an effective nanocarrier to efficiently deliver therapeutic agents like ICG.

CuS NPs attribute to be an incentive to many forms of cancer therapy. One such outstanding approach is that they are promising representatives of Photoacoustic Tomography (PAT). It is one of the most sort after cancer therapy strategies with a quite good penetration depth, better resolution etc. CuS NPs are also significant to be commendable candidates for PAI **(Figure 4c)** due to their high molar extinction coefficient, high photothermal efficiency and which brings about high conversion of light energy to heat energy. The prepared NC must display a number of notable traits that are crucial to its promotion as a highly photothermally effective material and to improving the approach of nanosystems in cancer nanotechnology. In addition, we also noticed that ICD is one of the type of tumor cell death activated by some of the chemo drugs, PTT and PDT and radiotherapies. ICD includes the DAMPs release from dying tumor cells that activate the tumor-specific immune response to eliciting the long-term efficacy to direct killing of tumor cells and antitumor immunity 50-52. Remarkably, dying tumor cells undergone ICD have been proposed to provoke antitumor effects. ICD markers of various DAMPs includes the cell surface exposure of CRT, ATP, and HMGB1 **(Figure 5d to g)**. In this work, we noticed the tumor cell death connected to ICD markers of the DAMPs exposed during photothermal ablation of tumor, and the mechanism by which they activate the immune system 53. Accordingly, the previous work also supported that the HCuSNPs-CpG NPs for cancer photothermal immunotherapy in a mouse model 30.

In conclusion, the stated NC initially were PEI coated CuS, which was successfully synthesized by a basic process in aqueous solution under the mechanism of electrostatic interaction. Here, the polymer or stabilizing agent or chelating agent was PEI, which is highly cationic in nature. Later, after the PEI coated CuS were synthesized, ICG and FA, the fluorescent imaging dye and targeting agent respectively were also successfully incorporated. The as-prepared NC were efficiently at 808 nm convert light energy to heat energy. The prepared NC with various analyses laid much emphasis on their stability, compatibility and good solubility. The NC also noted to have commendable photothermal conversion efficiency at 5 minutes upto a maximum temperature of 60 °C. The various stages of study revealed a good effect on the HeLa cells by interrupting on the tumor mechanism and showing great progress in obstruction of tumors. The study mainly focuses on the core NC i.e. CuS which was prepared to upgrade the properties it already possesses in order to make it more beneficial bio-medically. This is a preliminary attempt to study and analyse the reactive nature of CuS as an anti-cancer agent and to systematically bring out a NC that can add value and efficiency to the core. With great importance, it was also investigated that the NC upon NIR irradiation could help to ablate the tumor cells and ICD, concluding on superior treatment efficiency. The developed NC could also be applicable in PAI, which is an important headway in the field of cancer diagnosis. On account of this study, the formulated NC could be utilized not only as a therapeutic agent, but also as an image diagnostic agent for the treatment of cancer. Hence, this NC confirms to be a **“*theranostic agent*”** for cancer biomedical applications.

## Future Perspective

Research on CuS NPs for theranostic biomedical application is still infancy and several challenges are remains to overcome. Usually, NPs have a low photothermal conversion ratio and need high laser powers and longtime laser irradiation, which could damage to normal tissues and limits *in vivo* studies. Also, appropriate conjugation of targeting ligands to NPs surface with an impact on their size, morphology, cellular metabolism, and biosafety properties. Thus, CuS should be reasonably surface modified in order to improve the photothermal effect. Although, the CuS long-term toxicity and organ based toxicity should be clearly investigate with biocompatibility and possible role of antioxidant nature has also to be confirmed, but other necessary physiological and biochemical toxicity studies, should be conducted to further ensure the safety. However, other challenges also to overcome in order to apply clinical applications of this novel CuS NPs. CuS based NPs acted as a novel theranostic agent for cancer imaging and therapy. However, the incorporation of therapeutic and imaging agents within same NPs is a challenging task for the cancer heterogeneity nature of tumor microenvironment. Recently, **“*multifunctional theranostic agents*”** is highly emerging multidisciplinary efforts based on ultra-small CuS for molecular imaging and therapy with ample future prospects.

## Materials and Methods

### Materials Required

PEI coated CuS NPs was synthesized by a simple process in aqueous solution. All the required chemicals copper chloride (CuCl_2_), sodium sulfide (Na_2_S), polyethyleneimine (PEI), indocyanine green (ICG), Folic acid (FA) utilized in a process mentioned by the manufacturer. The cancer cell line selected was HeLa cell line and was procured from NCCS, Pune. All other chemicals and reagents used were of analytical grade. Ultrapure double distilled water (DW) was used during the course of the complete study.

### Formulation of CuS-PEI NPs Synthesis

To begin with the formation of the NC, the first step was the formation of PEI coated CuS NPs. CuCl_2_ (0.0176g), PEI (1.5 mL) and deionized water (70 mL) were mixed together and blue coloured solution is formed. This solution is made to stir for a time period of 30 minutes at room temperature. Later, Na_2_S (0.0124g) is added along with the stirring mixture and allowed to stir for another 10-15 minutes. At this point, the temperature of stirring is gradually increased up to 90 °C for a stirring period of 1 hour 30 minutes. Color change from dark brown color to dark green color is noted which is the end point of the reaction. The dark green color indicates the formation of CuS NPs and the solution is stored at 4 °C.

### Formation of CuS-PEI-ICG-FA NC

10 mL from the PEI coated CuS NPs solution is taken and then ICG (0.125 µL) is kept at magnetic stirrer at 780 rpm for 30 minutes. Later, FA (2.5 mg) was additionally added and allowed to stir for another 15 minutes. The dark green color is persistent and the solution is stored at 4 °C wrapped in an aluminum foil to avoid the photochemical reaction of ICG with the external surroundings.

### Method for Calculation of ICG Loading and Encapsulation Efficiency


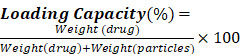

(1)


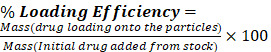

(2)

### Characterization Techniques of NPs

We examined and evaluate the material's structural properties by characterization methods. The different qualities, such as compound structure, compound variety, crystal structure and photoelectric properties, optical properties, and so on, are revealed by using a variety of material examination techniques and a wide range of standards. To determine its characteristics, the produced nanomaterial was detailed. The qualities displayed by the nanoparticles can be known or verified with the aid of characterization processes, which will further assist in selecting the uses of nanomaterials and increase their usability in various scientific and technological domains. The many techniques used to characterize the NP synthesis included:

### Spectrophotometric Analysis of NC

UV-Vis spectroscopy is the measurement of the attenuation of a light beam after it passes through a sample or after reflection from a sample surface. Absorption measurements can be obtained at a single frequency or over an all-inclusive spectral range, which may include the visible range, Ultra Violet (UV) range, or Near-Infrared (NIR) range. Infrared refers to the region beyond red, and ultra violet refers to the region beyond violet. It challenges the conventional wisdom that when a sample is exposed to light energy to the point where the energy gap between potential electronic transitions within a particle matches it, a small portion of the light energy will be ingested or absorbed by the atom, elevating the electrons to the higher energy state orbital. A spectrophotometer measures the amount of absorption a sample exhibits at different frequencies and plots the absorbance (A) vs frequency (λ), creating a spectrum, where λ_max_ is the frequency at which there is the highest absorption and the maximum intensity of absorption is known. For the NC, the NC sample is dispersed in water and observations of UV vis analysis is carried out under the range 300-900 nm. When light falls on the sample, each material absorbs specific range of light and thus the wavelength is noted.

### Dynamic Light Scattering and Zeta Potential Analysis of NC

Dynamic Light Scattering (DLS), the NC prepared since already in a dispersed phase initially, the samples were diluted with the prepared liquid during the process of synthesis and observed for DLS measurement of NC in triplicate at 25 °C using Malvern Zetasizer Nano ZS. The NP size polydispersity was expressed in Polydispersity Index (PDI). The zeta potential or surface charge analysis of the NC was carried out using Malvern Zetasizer Nano ZS at 25 °C. The results were obtained based on direction and velocity of particles under the influence of electric field.

### Fluorescence Spectrometry of NC

Fluorescence spectrometry is almost like absorbance spectrometry, anyway fluorescent compounds radiate light at a different wavelength that the wavelength of the irradiated light. At the point when fluorescent specie assimilates a photon, it passes from a ground state to an energized or excited state, after 1-10 nanoseconds, the fluorophore gets back to the ground state transmitting a photon. Due to the energy dispersal in the energized states, the energy of the transmitted or emitted photon is lower than the excitation photon. The interaction is repetitive (without photo bleaching) which implies the fluorophore can be energized cyclically. Like UV-vis spectrophotometer, the concentration of the analyte is corresponding with the intensity of the emission and can be determined. A relative low number of compounds show fluorescence and non-fluorescent molecules can fluoresce when labeled with a fluorophore.

### Storage Stability Analysis

ICG alone and NC were maintained at 4 °C in the dark for 30 days to evaluate the storage stability of the compounds. Both sample absorbance were measured after 0 and 30 days, and their percentage stabilities were estimated.

### Photothermal Performance Analysis of NC

To measure the photothermal performance of the as-synthesized CuS-PEI-ICG-FA NC, an 808 nm NIR laser was employed to be delivered perpendicularly through a quartz cuvette containing an aqueous dispersion (0.5 ml) of CuS-PEI-ICG-FA NC with a concentration of 100 µg/mL and water was used as a control. The NIR laser light source was equipped with a power of 1 W cm^-2^ under the 808 nm semiconductor laser device with a 5 mm diameter laser module. The temperature was measured using a thermocouple thermometer which was inserted into the aqueous dispersion perpendicular to the path of the laser light. The PTCE of the CuS-PEI-ICG-FA NC was determined according to the reported method 47.

### Haemolytic Analysis of NC

The haemolysis test was utilized to explore the lytic activity of the NC. It is carried out to attribute the blood compatibility level of the NC. This is highly used to understand how the NPs react *in vivo* experiments. Red blood cells (RBCs) were specifically obtained from human blood by eliminating the serum. To generate a clean supernatant, it was centrifuged five times at 4000 rpm after being further rinsed with 0.9 percent saline. RBCs were further diluted in PBS, and 0.3 mL of the diluted cell suspension was then combined with various amounts of PBS (negative control), deionized water (positive control), and NC suspension in 1 mL of suspension. The samples were agitated for a while, then held still for two hours. The mixes were then centrifuged at a speed of 4000 rpm, and the upper supernatants' absorbance was determined using UV-visible spectroscopy. The proportion of hemolysis was determined. Negative controls (RBCs in cushion just, without the presence of polymer) and positive controls (RBCs lysed with deionised water) were set up with a similar cell thickness.

### *In vitro* Photothermal Treatment Study

HeLa cells were chosen and treated in 96-well tissue culture plates at a density of 5×10^3^ cells/well in DMEM complete media for 24 hours in order to examine the photothermal ablation effect of the NC on tumor cell inhibition. The medium was then changed out for 100 L of brand-new DMEM complete media that included different formational CuS-PEI-ICG-FA NC concentrations ranging from 0 to 100 μg/mL. Then, for a further 5 minutes, the cell samples were subjected to an 808 nm near-infrared laser with a power density of 1 W cm^-2^. Each well was cleaned and 20 μL (5 mg/mL) of MTT solution was added after an additional 12 hours of incubation. The solution was removed from the plates and 200 μL of DMSO was then added after they had been incubated in the incubator for 4 hours. The plate was then given 10 minutes to shake on the table concentrator. Finally, the absorbance at 570 nm was measured using a microplate reader. The cell viability will be determined by the following equation: cell viability (%) = (mean of abs.value of treatment group/mean abs. value of control) × 100 %.

### Flow Cytometry Analysis

Using annexin V-FITC and propidium iodide (PI), the cells were stained to identify cancer cells that had undergone apoptosis or necrosis. In a nutshell, the cells had a CuS-PEI-ICG-FA NC pretreatment. The treated cells were suspended in 200 mL of binding buffer, followed by the addition of 10 mL of annexin V-FITC and 5 mL of PI, and incubation for 15 min at room temperature in the dark. After adding 300 mL of binding buffer to the cell suspension, the cells were flow-analyzed.

### Photoacoustic Imaging (PAI) Property of NC

Photoacoustic imaging (PAI) is also called optoacoustic imaging is a recent technology that encourages the cancer detection and treatment strategies. It is a highly advantageous study that provides high emphasis to the structural, molecular and functional information during the pre-clinical studies itself. Imaging methods assume a huge part in cancer treatment, from exact arranging and directing to assessment of viability. Specifically, PAI has shown potential in supporting treatments by giving successive observing of tumor utilitarian properties, for example, changes in tumor vasculature previously, during, and after therapeutic procedures. A multi-mode PA imaging technology thermal imager (Testo 875, German make) was utilized to observe the PA imaging property of the as-prepared NC (CuS-PEI-ICG-FA). The NC were taken in various concentrations (0, 25, 50, 75, and 100 µg/mL) for the study. Upon an exposure of 808 nm NIR, the respective PA signals were analyzed and were detected with aid of a transducer.

### Ethics Statement for *In vivo* Experiments

All *in vivo* animal testing was done in accordance with guidelines authorized by the Institutional Review Board. Mice were used in animal experiments after receiving prior clearance from the institutional animal ethical committee (IAEC), which was established by the Animal House Division. The experiments were conducted in accordance with the CPCSEA's strict standards and recommendations. According to CPCSEA norms, the experiments and animal handling were done. The techniques followed the standards for the moral treatment of animals. All balb/c mice weighing 20 g were obtained from the Kerala Veterinary and Animal Sciences University, Animal Centre, and maintained in suitable experimental conditions such as room temperature of 24 °C and 40-60% of relative humidity while on a regular 12 h light-dark cycle. Animal usage was officially permitted by the IAEC, and *in vivo* animal experiments were carried out in agreement with the IAEC of the university. Prior to the *in vivo* trial, the test animals underwent a minimum of three days of acclimatization.

### *In vivo* Antitumor animal Experiment

Female Balb/c mice (4-5 weeks old) were used in animal investigations. They were acclimated at 25 °C and 55% humidity under natural light/dark circumstances with the approval of the ethics committee. For the mouse tumor animal model, HeLa cells were employed, and they were kept alive in DMEM. After administering 45 mg/kg of pentobarbital sodium intraperitoneally to anaesthetize the mice, 1×10^6^ cells/mL of cells suspended in 200 μL of PBS were injected into the mice to create subcutaneous tumors. The animals were randomly assigned to different treatment groups after 15 days, when the tumor had grown to between 150 and 200 mm in diameter. Photothermal investigations were subsequently carried out when the tumor had reached the aforementioned size. The mice were randomly divided into four groups (n=5 for each group) and treated with the following experimental conditions: (1) Blank PBS as control group (2) injection of NC alone (3) 808 nm NIR laser exposure alone (4) Test group: PBS solution of NC was intratumorally injected into the tumor mice which were exposed to 808 nm NIR laser (1 W/cm^-2^) for 5 min. The local maximum temperature was recorded on an infrared thermal imaging camera (Testo, Germany). After these treatments, the tumor size was measured by a caliper every three day and calculated as volume = (tumor length) × (tumor width)^2^/2. Relative tumor volumes were calculated as V/V_0_ (V_0_ is the tumor volume when the treatment was initiated). The body weights of the mice were also measured, and the findings were shown as a function of time. Mice (n = 3) that had been intravenously injected with NC in PBS were used in the experiment to assess the half-life of blood circulation *in vivo*. 15 μL of mouse blood was taken at various intervals up to 24 h after the post-injection and dissolved in 985 μL of physiological saline that contained 10 mM EDTA as an anticoagulant. The sample's concentration was established. On the basis of a single compartment pharmacokinetic model, the curve for blood circulation half-life was fitted.

### Immunogenic Cell Death Marker Detection

Using an ATP assay kit, the amount of adenosine 5′-triphosphate (ATP) released extracellularly was determined. On a nutshell, tumor cells were plated in six-well cell culture plates and treated in various ways for 3 hours with NPs. After being collected, the culture supernatant was centrifuged at 12 000 g for 10 min. The supernatant was then put into tubes to check for the release of ATP. Using a multiscan spectrum (Thermo Fisher Scientific & Co.) and 100 μL of ATP detection buffer, the luminescence from 20 μL of the sample was evaluated. The ATP level was subsequently adjusted in accordance with the protein concentration. ELISA was used in a second ICD marker assay for HMGB1 release in tumor cells. In six-well plates, 3×10^6^ cells were planted each well. Cells were exposed to NPs with various treatments for 3 hours after cells had been incubated for 24 hours. ELISA assay kit was used to find HMGB1 expression. Cells were then seeded at a density of 1×10^5^ per well in 24-well plates and cultivated for an additional 24 hours in order to detect CRT expression. After a further 4 hours of incubation, the cells were subjected to PBS, NC, NIR, and NC+NIR laser treatments. Individual primary antibodies against CRT were incubated with the treated cells for 30 min. After that, a fluorescence microscope was used to quantify and analyse the CRT exposure.

### Histopathological Examination

After 15 days of treatment, mice from each group were put down, and their organs (heart, liver, spleen, lung, and kidney) were removed and preserved for histological examination in 4% neutral buffered formalin. The organs were then gradient-dehydrated using various ethanol and xylene concentrations. The samples were subsequently cut into slices and submerged in liquid paraffin for H&E staining. Finally, a microscope was used to look at each organ's morphological properties (Olympus, Japan).

### Long-Term Toxicity Assessment *in vivo*

NC suspension was injected into healthy Balb/c mice, and the mice were euthanized at different intervals after the injection. Each mouse had blood drawn from them three times: on the first day, the seventh day, and the fifteenth day. This blood was used for the complete blood panel analysis and blood biochemistry testing. The levels of ALT, ALP, and AST in the serum were used to assess liver function. By measuring the levels of BUN, Cr, CAT, GSH-PX, and SOD, kidney function was evaluated. To confirm the long-term toxicity *in vivo*, additional parameters like GLU, CHOL, and MCV were also assessed.

### Statistical Analysis

Statistical Program for Social Sciences (SPSS) software was used as needed for statistical analysis. With the exception of the mentioned tests, all data are presented as means with standard deviation, and statistically significant differences are indicated in the figures by asterisks at *p<0.05 and **p<0.01, respectively.
