# Supplementary material for: Ultra-small NIR-Responsive Nanotheranostic Agent for Targeted Photothermal Ablation Induced Damage-Associated Molecular Patterns (DAMPs) from Post-PTT of Tumor Cells Activate Immunogenic Cell Death
Source: Nanotheranostics. 2023 Jan 1;7(1):41–60. doi: 10.7150/ntno.76720 (PMC9760365; doi:10.7150/ntno.76720)
Supplement: Supplementary file 1 — Supplementary figures. [file ntnov07p0041s1.pdf]

Supplementary Materials

Ultra-small NIR-Responsive Nanotheranostic Agent for Targeted Photothermal Ablation Induced Damage-Associated Molecular Patterns (DAMPs) from Post-PTT of Tumor Cells Activate Immunogenic Cell Death

Shankar Sobhana[1¶], Namratha Partha Sarathy[1¶], Laxmanan Karthikeyan[1¶], Krishnamurthy Shanthi[2] Raju Vivek[1]*

[1]Bio-Nano Therapeutics Research Laboratory, Cancer Research Program (CRP), Department of Zoology, Bharathiar University, Coimbatore-641 046, TN, India.

[2]Department of Biochemistry, Prof. Dhanapalan College of Science and Management, Chennai, India.

[¶]These authors contributed equally to this work

Corresponding author: Raju Vivek: vivekr@buc.edu.in. Bio-Nano Therapeutics Research Laboratory, Cancer Research Program (CRP), Department of Zoology, Bharathiar University, Coimbatore-641 046, TN, India.

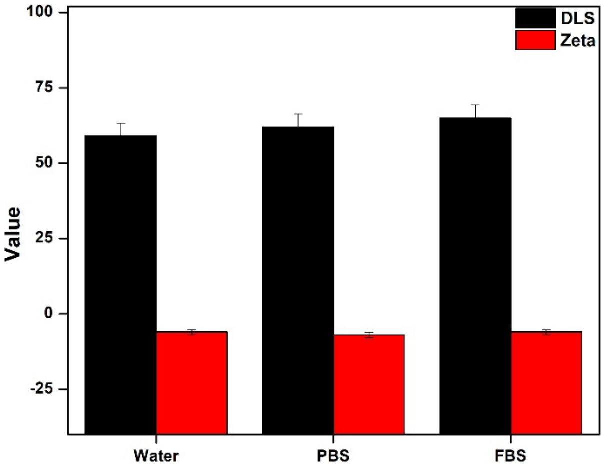

Figure S1: DLS and zeta pontential of the NC in various biological medium.

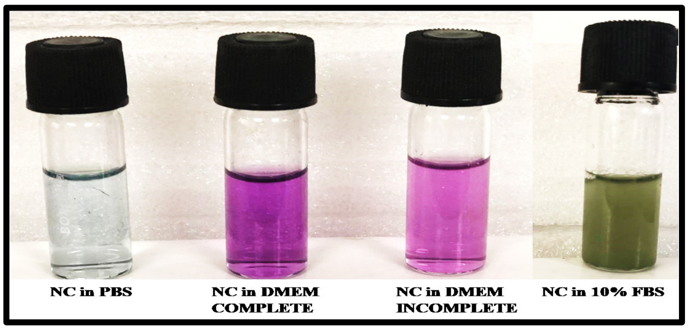

Figure S2: Stability of NC in various biological medium.

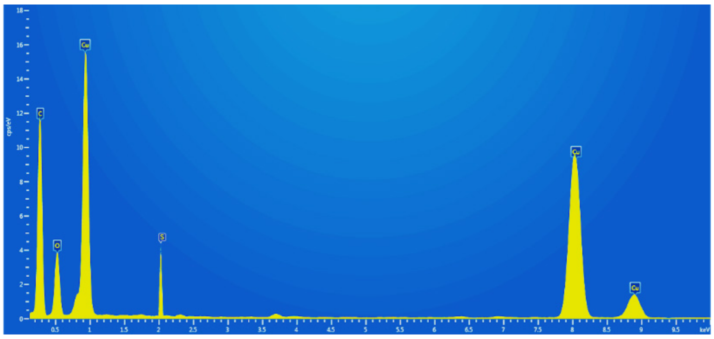

Figure S3: EDX elemental analysis of the NC for the confirmation of Cu and S.

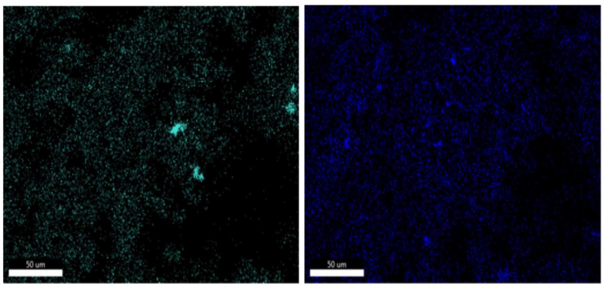

Figure S4: Corresponding elemental mapping of Cu, and S in the NC.

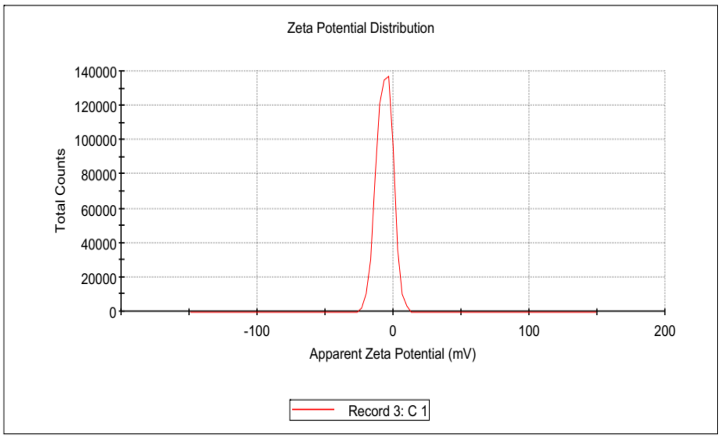

Figure S5: Zeta potential of the NC for the surface charge analysis.

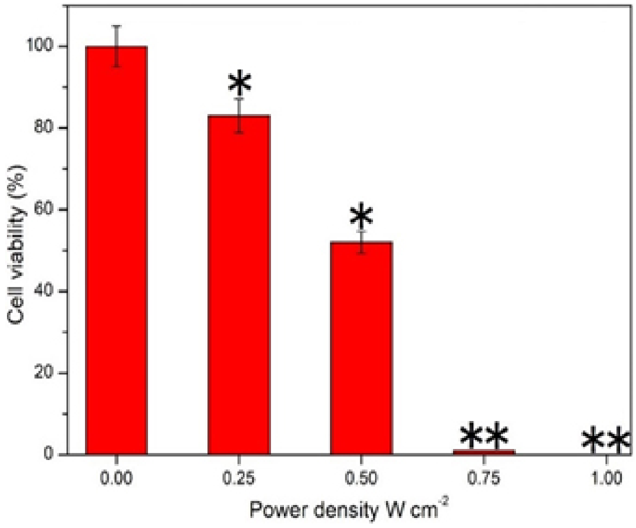

Figure S6: Cell viability of NC treatment under 808 nm NIR light with various density.

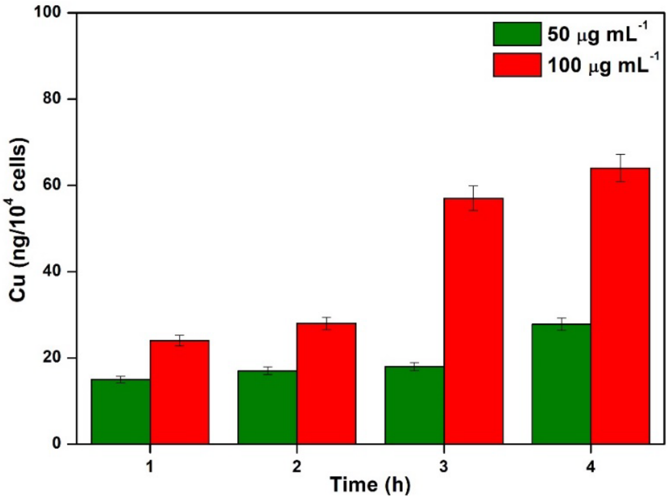

Figure S7: Quantitative in vitro cellular uptake study of the NC analyzed by inductively coupled plasma mass spectrometry (ICP-MS) measurement.

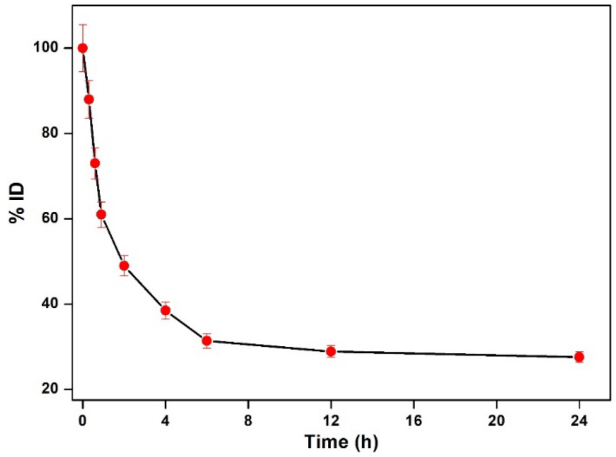

Figure S8: Blood circulation profile of NC in various time points.

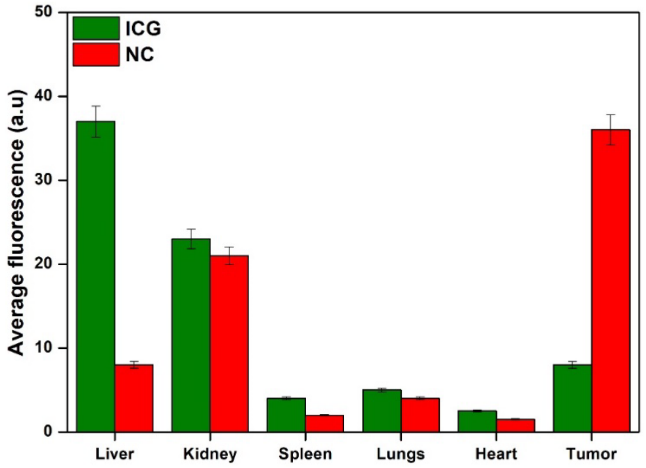

Figure S9: Biodistribution of free ICG and NC in mice determined by the ICG FL from diluted tissue lysates. The data are shown as mean±SD (n=3).

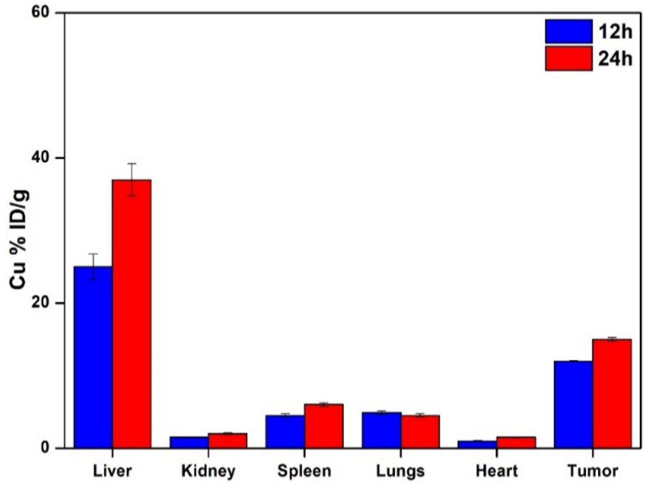

Figure S10: After being treated with NC for various time, the biological distribution of Cu accumulation in each organ: liver, kidney, spleen, lungs, heart, and tumor. The data are represented as mean ± SD, n=6 mice per group. Data are mean ± SD.
